# Characterization of the PH1704 Protease from *Pyrococcus horikoshii* OT3 and the Critical Functions of Tyr120

**DOI:** 10.1371/journal.pone.0103902

**Published:** 2014-09-05

**Authors:** Dongling Zhan, Aixi Bai, Lei Yu, Weiwei Han, Yan Feng

**Affiliations:** 1 Key Laboratory for Molecular Enzymology and Engineering of Ministry of Education, School of Life Science, Jilin University, Changchun, China; 2 College of Food Science and Engineering, Jilin Agricultural University, Changchun, China; 3 State Key Laboratory of Microbial Metabolism, College of Life Science and Biotechnology, Shanghai Jiao Tong University, Shanghai, China; Russian Academy of Sciences, Institute for Biological Instrumentation, Russian Federation

## Abstract

The PH1704 protease from hyperthermophilic archaean *Pyrococcus horikoshii* OT3 is a member of DJ-1/ThiJ/PfpI superfamily with diverse functional subclasses. The recombinant PH1704 was efficiently purified and was systematically characterized by a combination of substrate specificity analysis, steady-state kinetics study and molecular docking research. The homogeneous protease was obtained as a presumed dodecamer with molecular weight of ∼240 kDa. Iodoacetamide strongly inhibited the peptidase activity, confirming that Cys100 is a nucleophilic residue. The recombinant protein was identified as both an aminopeptidase and an endopeptidase. Experimental data showed that L-R-amc was the best substrate of PH1704. Structural interaction fingerprint analysis (SIFt) indicated the binding pose of PH1704 and showed that Tyr120 is important in substrate binding. Kinetic parameters *K*_cat_ and *K*_cat_ */K*_m_ of the Y120P mutant with L-R-amc was about 7 and 7.8 times higher than that of the wild type (WT). For the endopeptidase Y120P with AAFR-amc, *K*_cat_ and *K*_cat_ */K*_m_ is 10- and 21- fold higher than that of WT. Experimental data indicate the important functions of Tyr120: involvement in enzyme activity to form a hydrogen bond with Cys100 and as an entrance gate of the substrate with Lys43. The results of this study can be used to investigate the DJ-1/ThiJ/PfpI superfamily.

## Introduction

*Pyrococcus horikoshii* is a thermophilic *archaean* with an optimal growth temperature about100°C and grows at a greater sea depth than the other archaea [1]. The study of *Pyrococcus horikoshii* can provide insight into possible mechanisms used to endure high temperatures and high-pressure environmental conditions.

Enzymes derived from microorganisms growing at such extreme temperatures can be used in biotechnology as highly thermostable biocatalysts [1]. The PH1704 protease from *Pyrococcus horikoshii* OT3 is a hyperthermophilic enzyme that belongs to the DJ-1/ThiJ/PfpI superfamily [2]. The DJ-1/ThiJ/PfpI superfamily is diverse and large, with representatives in nearly all organisms [3]. One of its members, the human protein DJ-1, has recently been reported to cause certain types of early-onset Parkinsonism [4]. Thus, an increasing number of studies on this superfamily are being conducted. Despite this growing interest, few members of this superfamily have been biochemically characterized. For example, heat-shock protein 31(Hsp31) was characterized as a chaperone and a peptidase [5]–[7], *Pyrococcus furiosus* protease I (PfpI) exhibited protease/peptidase activity [8], [9], PH1704 is regarded as a protease [2], [10], and *Escherichia coli* YhbO is involved in the response to hyperosmotic or acid stress [11].

**Figure 1:**
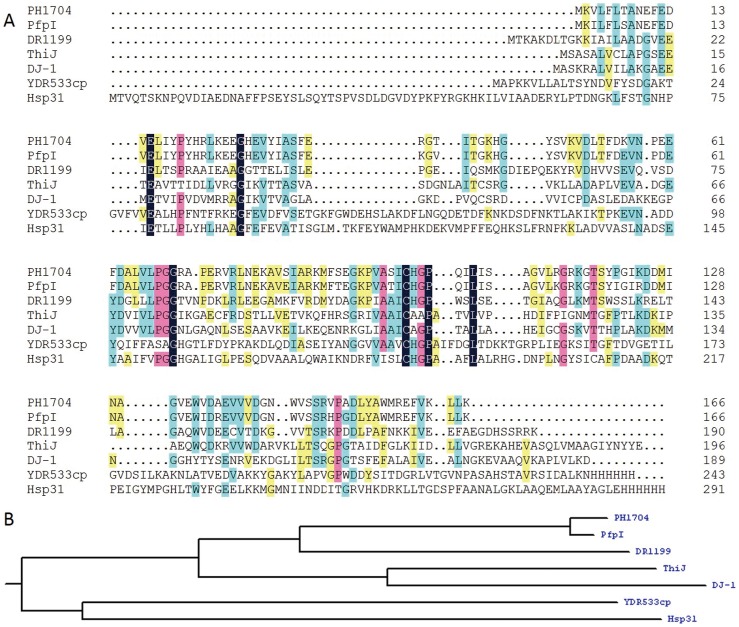
Sequence alignment and phylogenetic tree of PH1704. A, Sequence alignment of PH1704 with other members in the DJ-1 superfamily. PfpI (gi/18978091) 90% sequence identify with PH1704; DR1199 (PDB Id 2VRN), 46% sequence identify with PH1704; ThiJ from *E. coli* (gi/190906193), 27% sequence identify with PH1704; YDR533Cp (PDB Id 1RW7) 25% sequence identify with PH1704; Human DJ-1 (PDB Id 1J42), 24% sequence identify with PH1704; Hsp31 (PDB Id 1N57) 19% sequence identify with PH1704. The color purple represent for similar sequence, and color green represent for identical sequence. B, a phylogenetic tree of the DJ-1/ThiJ/PfpI superfamily and several closely related sequences. The tree contains three more closely related clades: Hsp31 is in the Hsp31 family class I, whereas PH1704 and PfpI are in the Hsp31 family class III, with the homolog of the same family.

Members of the DJ-1/ThiJ/PfpI superfamily have two common characteristics: (i) they share a low sequence identity (except for PH1704 and PfpI, which have a 90% sequence identify, as shown in Fig. 1A) and the similar α/β sandwich tertiary structures [12]–[15]; (ii) they are characterized as oligomers [2] from dimers to trimers, hexamers, and higher forms. The two characteristics can be crucial to either stability or physiological activity of these proteins [2], [16], [17].

**Figure 2:**
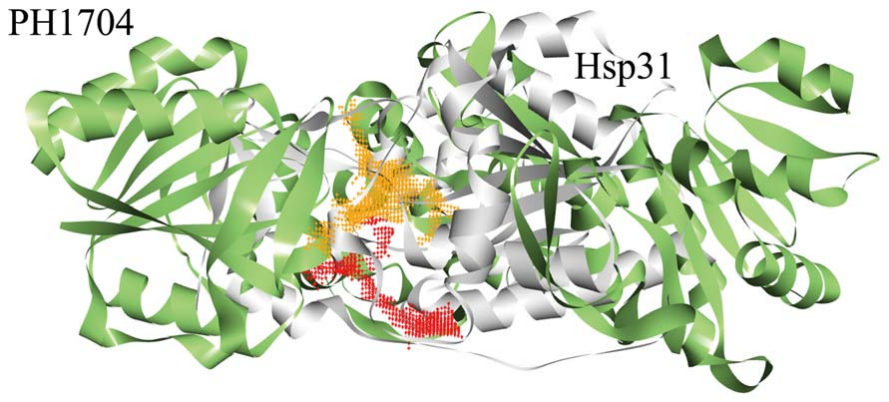
The active pocket compare with PH1704 (green) and Hsp31 (Gray). The color orange dot represent for the active pocket for PH1704 (the active pocket size of PH1704 is 1622.1 Å^3^, calculated by CASTp server [37] with 1.4 Å), color red dot represent for the active pocket for Hsp31 (the active pocket size of is 980 Å^3^, calculated by CASTp server with 1.4 Å). It can be concluded that the active pocket size of PH1704 is larger than that of Hsp31.

Among the DJ-1/ThiJ/PfpI superfamily, only three proteins exhibit peptidase activity: PH1704 (PDB Id 1G2I) [2], PfpI [8], [9] and Hsp31 (PDB Id 1N57) [5]–[7]. Quigley et al. recently proposed clustering the members of this superfamily, according to their superstructure, into three subfamilies [7]: the Hsp31 family class I, Hsp31 family class II, and Hsp31 family class III. A phylogenetic tree of the DJ-1/ThiJ/PfpI superfamily and its closest related sequences was constructed based on multiple sequence alignment (Fig. 1). The tree contains three more closely related clades: Hsp31 is in the Hsp31 family class I, whereas PH1704 and PfpI are in the Hsp31 family class III, with the homolog of the same family, Dr1199 [18] is classified in the MEROPS peptidase database (http://merops.sanger.ac.uk) as a non-peptidase. These three clades exhibit distinct enzymatic specificities, as described below. In addition, these clades show distinct active site pockets although insertions and deletions (gaps at the alignment level) are not considered in the tree reconstruction algorithm. The amino acid sequence of PH1704 shares a 90% identity with that of PfpI, suggesting that the physiological character of PH1704 is the most similar to PfpI during peptide degradation [8], [9]. PfpI is an endopeptidase and can degrade large proteins, including azocasein and gelatin, at 85°C [9]. However, the three-dimensional (3D) structure of PfpI remains unknown. Research on Hsp31 family class III only includes the preliminary biochemical characterization of PfpI and the structural determination of PH1704. Hsp31 also exhibits aminopeptidase activity. Although the sequence homology of Hsp31 and PH1704 is only 19% and their oligomerization states differ, the spatial conformation of the catalytic triad is similar. The substrate-binding cavity of PH1704 is larger than that of Hsp31 (Fig. 2). This difference can determine different substrate specificities and thus distinguish the substrate specificity of PH1704 from that of Hsp31.

In this study, the first efficient purification procedure of PH1704 is described. Preliminary enzymology characterization was conducted by determining the optimal reaction temperature. A further insight into catalytic properties resulted from an aminopeptidase cleavage preference study and kinetic investigation. Structural interaction fingerprint (SIFt) [19], [20] showed the most critical anchor residues responsible for substrate binding. From the kinetic parameters of the mutant Y120P, we can conclude that the special site is closely relevant to substrate binding and nucleophilic attack of Cys100. The experimental data also indicated that Tyr120 and Lys43 act as entrance gates to guiding the substrates to slide inward.

## Materials and Method

### Reagent, strains, and plasmids

Restriction enzymes were purchased from Promega (Madison, WI). *Pfu* DNA polymerase was supplied by Stratagene (Madison, WI). Ampicillin and isopropyl thio-β-D-galactopyranoside (IPTG) were obtained from TaKaRa Shuzo (Otsu, Shiga, Japan). The 7-amino-4-methylcoumarin (amc)-linked substrates L-D-amc, L-S-amc, L-V-amc, L-A-amc, L-F-amc, L-R-amc, and hippuryl-R were purchased from GL Biochem (Shanghai, China). The His-Tag™ monoclonal antibody and the rabbit anti-mouse peroxidase-conjugated secondary antibody were purchased from Merck (San Diego, CA). Other chemicals used were reagent-grade. The pET-15b vector and the *E. coli* strain were purchased from Novagen (Madison, WI).

### Construction of expression vectors

Genomic DNA was obtained from *P. horikoshii* OT3 by using the QIAGEN DNeasy tissue kit. Based on the DNA sequence of PH1704 (GenBank accession no. O59413), two primers, 5′-GGCAGCCATATGGTGAAGGTACT-3′ and 5′-GCAGCCGGATCCTCATTTAAGTA-3′, were synthesized to amplify the gene encoding PH1704 by polymerase chain reaction (PCR). The underlined bases correspond to the restriction enzyme sequences of NdeI/BamHI, respectively. PCR conditions involved initial denaturation at 95°C for 10 min, followed by 30 cycles of 95°C for 1 min, annealing at 55°C for 1 min, and extension at 72°C for 30 s. After purification with a QIAquick PCR purification kit, the PCR product was digested with NdeI/BamHI and ligated to the same restriction sites of the pET-15b vector to generate the expression plasmid pET15b-PH1704.

### Expression and purification of the recombinant PH1704

The expression vector with the target gene (pET15b-PH1704) was transformed into *E. coli* BL21 (DE3). Positive transformants were cultured in Luria-Bertani medium with 100 g/mL ampicillin to an absorbance at 600 nm of 0.5 to 0.8 and then induced with 1 mM IPTG for 12 h at 26°C. Cells were harvested by centrifugation at 5000 rpm for 30 min and disrupted by sonication in a 50 mM Tris-HCl buffer (pH 8.0). Crude bacterial extracts were subjected to heat incubation at 80°C for 15 min. The supernate was collected by centrifugation and then filtered through a 0.45 µm membrane to remove the residual cells and the heat-induced aggregated proteins. Subsequently, the supernate was loaded onto a Sephacryl S-200 chromatography column. Proteins were eluted from the column with the 50 mM Tris-HCl buffer (pH 8.0) supplemented with 150 mM NaCl. The purity of the protease was analyzed by non-denaturing.

### Non-denaturing PAGE, zymograms, and western blot

Non-denaturing PAGE was modified with the Laemmli method [21]. Electrophoresis was implemented using a 10% (w/v) polyacrylamide resolving gel and a 5% (w/v) polyacrylamide stacking gel. After electrophoresis, proteins in the gel were visualized with Coomassie Brilliant Blue. For zymogram analysis, gelatin (0.1%) copolymerized with the acrylamide gels was used. Electrophoresis was performed at 4°C at a constant voltage of 100 V. The gels were incubated in HEPES (50 mM) buffer for 2 h at 4°C and then in Tris-HCl buffer (pH 8.0) for 3 h at 70°C. The gels were finally soaked in 20% ice-cold trichloroacetic acid for 1 h and stained with Coomassie Brilliant Blue. Proteolytic activity was indicated by the formation of clear zones in the gel containing gelatin. For western blot, the proteins were separated by 10% non-denaturing PAGE and transferred onto a polyvinylidene fluoride membrane. The membrane was probed with the His-Tag TM monoclonal antibody (Merck, San Diego, CA) at a dilution of 1∶1000 in tris-buffered saline (TBS) for 2 h, followed by the rabbit anti-mouse peroxidase-conjugated secondary antibody (Merck, San Diego, CA) at a dilution of 1: 5000 in TBS for 1 h. The blot was developed using a 3,3-diaminobenzidine tetrahydrochloride solution.

### Steady-state kinetics assay of the wild-type and mutant PH1704

The kinetic parameters of PH1704 and mutants were determined by the cleavage of amc of L-R-amc and AAFR-amc. Each assay tube contained 200 µL of substrate stock (100 mM in dimethyl sulfoxide; DMSO), 50 µL of diluted enzyme sample, and 250 µL of 50 mM sodium phosphate buffer with a pH of 7.5. The assay tubes were incubated at 358 K in a thermocycler for 60 min, chilled on ice, and centrifuged. Fluorescence was measured at 360 nm ± 40 nm excitation and 460 nm ± 40 nm emission. Measured fluorescence units were converted to picomoles of L-R- amc/AAFR-amc released using a standard curve prepared with known amc dilutions in 5% DMSO in 50 mM sodium phosphate, pH 7.5. Initial steady-state velocities were monitored with a substrate concentration ranging from 0.5 µM to 400 µM. All measurements were performed in triplicate. The kinetic parameters were determined from the hydrolysis rates by fitting the Hill equation (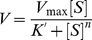) to the data points, using the nonlinear least-squares method [22]. The standard error for each parameter was estimated from curve fitting.

### Characterization of recombinant PH1704

The optimal pH was determined using a 50 mM Britton–Robinson wide-range buffer with pH ranging from 4 to 11 at a 0.5 pH scale interval by using AAFR-amc as a substrate [23]. The optimal reaction temperature was determined by incubation of reaction mixtures at varying temperatures from 30°C to 95°C at 5°C intervals.

The substrate spectrum of the recombinant PH1704 was determined using different kinds of fluorogenic substrates under standard assay conditions. Substrates L-S-amc, L-D-amc, L-V-amc, L-A-amc, L-F-amc, and L-R-amc were applied. Hippury L-R was also used to determine the carboxypeptidase activity, as previously described [24], [25].

The effect of metal ions on protease activity was determined by adding the following metal ions: K^+^, Na^+^, Mg^2+^, Ca^2+^, Mn^2+^, Cu^2+^, Zn^2+^, Fe^3+^, Ni^2+^, and Co^2+^. The enzyme was incubated with each of the metal ions at 25°C for 30 min. These ions were investigated at a final concentration of 0.2, 1.0, and 5.0 mM, respectively. The activity was assayed using AAFR-amc as a substrate under standard conditions. The protease activity of the enzyme without any additional metallic ion was defined as 100%.

The effects of inhibitors on protease activity were determined using various inhibitors [(phenylmethylsulphonyl fluoride (PMSF), diethylpyrocarbonate (DEPC), and iodoacetamide (IAA)] at final concentrations of 1, 5, and 10 mM. The enzyme was incubated with each inhibitor at 25°C for 30 min in 50 mM of Tris-HCl buffer (pH 8.0) in the darkroom. Enzyme assay was used to measure the substrate AAFR-amc under standard conditions. The protease activity of the enzyme without the addition of inhibitors was defined as 100%.

### Structural interaction fingerprint (SIFt) analysis

SIFt represents a class of binary fingerprints directly related to protein–ligand interactions and can indicate the binding pose of protein [19], [20]. In the present study, nine bits (any, backbone, sidechain, polar, hydrophobic,H-donor,H-acceptor,aromatic and charged) were used to describe these interactions.

Protein residues are grouped into four classes: polar, hydrophobic, aromatic, and charged. For each atom of a ligand, the residues within cut-off range are selected. The occurrence of an interaction is determined by atom–atom distance, type of atoms/residues, and appropriate angle in case of hydrogen bonds. So an average SIFt may by generated for the population of ligands and/or receptors. And then the sequentially recalculated for every amino acid in the population of ligands docked into each receptor, comparing with the alternative complexes.

AutoDock 4.2[26], [27] was used to perform molecular docking. Results were clustered according to the 1.0 Å root mean square deviation (RMSD) criteria. All torsion angles for each compound were considered flexible. The grid maps representing the proteins in the actual docking process were calculated by AutoGrid. The 3D structure of PH1704 was downloaded from the Protein Data Bank (PDB), whereas the 3D structure of PfpI was constructed by the SWISS-MODEL (http://swissmodel.expasy.org/) and then checked by Procheck, Errat, and Prove. The 29 ligands were drawn with ChemDraw 3D and then optimized with Gaussian 03 at the B3LYP 6–31G* level [28], [29]. The two proteins (PH1704 and PfpI) and 29 ligands were changed into the PDBQT format with AutoDock tools. The grid size for docking was 56 Å×56 Å×56 Å.

## Results

### Expression and purification of recombinant PH1704

**Figure 3:**
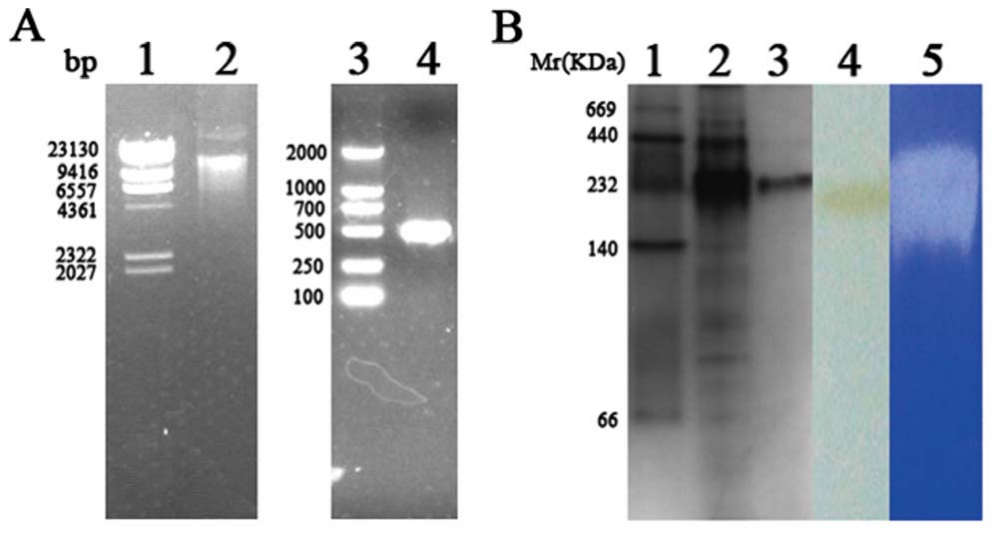
Cloning, expression, validation and proteolytic activity of PH1704. A, Lane 1: Hind III Marker; lane 2: Pyrococcus horikoshii genome; Lane 3: DL2000 Marker; lane 4: PCR product using P. horikoshii genome as template. B, Lane 1: High molecular weight protein marker; lane 2: PH1704 crude extract after heated at 80 oC for 15 min; lane 3: The purified recombinant PH1704 protease on non-denaturing gel; lane 4: Western blotting analysis of the purified PH1704; lane 5: enzyme assays of proteolytic activity on 10% Non-denaturing PAGE with 0.1% gelatin;lane 6: negative control on 10% Non-denaturing PAGE with only 0.1% gelatin substrate.

As shown in Fig. 3A, the desired fragment of the *P. horikoshii* PH1704 gene was amplified by PCR from the genomic DNA of OT3. The gene encoding PH1704 was inserted into the expression vector pET-15b. The resulting plasmid was transformed into *E. coli* by electroporation and plated on LB medium containing ampicillin (100 µg/mL). Correct insertion and reading frame of PH1704 were confirmed by DNA sequencing. The *E. coli* transformant was cultured, and PH1704 expression was induced with 1 mM IPTG. After 12 h of induction at 26°C, the culture medium was centrifuged, and the cells were collected. The cell lysate was then subjected to 15 min of heat incubation at 80°C to remove most sensitive proteins from *E. coli.* Size-exclusion chromatography (Sephaeryl S-200) was performed to eliminate contaminant proteins and other macromolecules. Affinity chromatography was not suitable for this enzyme because recombinant PH1704 can be precipitated in the present of nickel. About 50.2 mg of purified recombinant PH1704 was obtained from 1 L of culture cell. The total purification efficiency was 8.01- fold, as indicated in Table 1. The purified protein was resolved as a single band with a molecular weight of 240 KD by Non-denaturing PAGE and verified by western blot anaylsis using anti-His tag antibody (Fig. 3B, lane3 and 4). The higher form of recombinant PH1704 exhibited higher activity towards gelatin (Du *et al*., 2000). The purified 660 KD protein aggregated to larger assembly (>200 KD) at 1.0 mg/ml [8], which is consistent with the result in this study that the 240 KD recombinant PH1704 constituted most protein (Fig. 3B, lane 2). We obtained the single 240 KD protease for higher activity and convenience. The purified recombinant PH1704 was presumed a dodecamer based on the molecular weight of the gel. Most protein purification protocols require multiple steps to obtain highly purified products. These steps include complex procedures necessary to transfer products between different conditions for various purification techniques. An ideal purification protocol includes high-yield and high-purity requirements with few steps and a simple design, without the loss of enzymatic activity. Halio et al. expressed PfpI in *E. coli* and purified the protein with DEAE-Sepharose, hydroxyapatite, Pheny L-Sepharose, Mono Q and gel filtration chromatography at different stages [9]. They obtained high-purity proteins, however, the process entailed high costs. The present study proposes a low-cost method of preparing high-purity thermophilic protein for further functional studies.

**Table 1:** Purification of the recombinant PH1704 from *Pyrococcus horikoshi* OT3.

Steps	Total Protein (mg)	Total activity (U)	Specific activity (U/mg)	Yield (%)	Fold purification
Crude extract	3048.9±209.7	N	N	-	-
Heat incubation	672.5±58.2	1670.4±111.4	2.48±0.12	100	1.03±0.04
Sephacryl S-200	50.2±4.3	997.474±60.1	19.87±0.56	59.71±5.52	8.01±0.27

Each experiment was repeated three times.

### Optimal catalytic temperature, pH, and inhibitors of PH1704 activity

**Figure 4:**
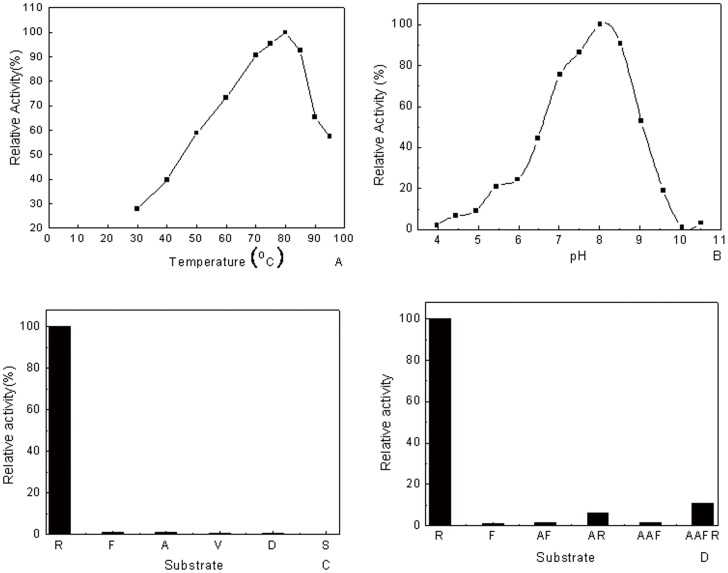
Effects of pH and temperature on the recombinant PH1704's activity. A, The temperature activity profile; B, pH dependence reactions catalyzed by PH1704; Substrate specificity of recombinant PH1704. The aminopeptidase activity towards L-R-amc was defined as 100%; C, The aminopeptidase substrate specificity; D, The endopeptidase substrate specificity.

Zymogram analysis with gelatin was conducted to verify the finding that PH1704 could hydrolyze gelatin as a protease [2]. A clear hydrolytic zone on the gel confirmed the aforementioned finding (Fig. 3B, lane 5). To determine the optimal catalytic conditions, AAFR-amc was used as the endopeptidase substrate for experimental convenience, as reported [8], [9]. As shown in Fig. 4, the highest activity of PH1704 was observed at approximately 80°C, pH 8.5.

Many metal ions influence protease activity [30], [31]. The effects of 10 kinds of metal ions on the protease activity were investigated (Table 2). Table 2 reveals that all cations negatively affected the hydrolysis activity. Ni^2+^ reached a maximal inhibition of 85% and could precipitate the protein at higher concentrations (1 mM and above). Zinc decreased the catalytic activity to 76% at 1 mM but was essential for Hsp31 activity [32].

**Table 2:** Effects of metal ions on enzyme activity (%).

Metal ion	Relative activity (%)		
	Concentration (mM)		
	0.2	1.0	5.0
Control	100	100	100
K^+^	101.18±7.91	95.09±6.87	81.45±4.2
Na^+^	86.77±4.77	91.33±5.62	71.97±4.66
Mg^2+^	90.15±6.16	102.79±7.66	89.66±5.91
Ca^2+^	68.99±3.89	80.59±4.87	71.18±3.86
Mn^2+^	73.03±4.73	57.37±3.78	19.38±0.94
Cu^2+^	43.17±2.68	19.73±1.09	ND
Zn^2+^	37.42±1.99	23.61±1.22	ND
Fe^3+^	72.77±3.57	31.60±1.64	ND
Ni^2+^	15.52±1.02	ND	ND
Co^2+^	25.28±1.53	ND	ND

ND, the enzyme was precipitated by the ions at the above concentrations. Thus, the activity could not be determined. Each experiment was repeated three times.

Cys100 is highly conserved among the superfamily (seen from Fig. 1A), although human DJ-1 does not function as an enzyme (seen from Fig. 1). It is reported the Cys106 of human DJ-1 which is equivalent to Cys100 in PH1704 is the most sensitive residue under the oxidative environment [33], [34]. Cys185 of Hsp31 was identified as nucleophilic site by inhibition and mutational test [5]–[7]. Du speculated that Cys100 was the nucleophile residue responsible for the protease activity from crystallographic data [2]. To validate this hypothesis, we determined the protease activity in the presence of various inhibitors by using AAFR-amc as a substrate. As shown in Table 3, addition of PMSF has no significant effects on the protease activity of PH1704, indicating that serine near the active site is not critical to the catalysis. DEPC as a histidine reagent [35] exhibited a slight inhibitory effect on the activity. The substrate-binding pocket likely prevented DEPC from accessing the active site, thereby preserving the activity. The inhibition efficiency of sulphydryl alkylating agent iodoacetamide (IAA) was enhanced to nearly 90% with an increase in concentration. This observation indicates that IAA covalently modifies Cys100 and inhibits protease activity. This study confirms the structure observation of PH1704. Cys100 is essential as a nucleophilic agent for the catalysis of both PH1704 and Hsp31 [5], [6], [7], [32].

**Table 3:** Effects of inhibitors on enzyme activity (%).

Compound	Relative activity (%)		
	Concentration (mM)		
	1.0	5.0	10.0
Control	100	100	100
DEPC	97.67±6.45	101.05±7.12	88.43±5.34
PMSF	86.36±4.93	73.02±3.08	70.04±3.21
IAA	52.6±3.03	23.5±1.06	12.3±0.88

Each experiment was repeated three times.

### Substrate specificity analysis

Two reports from Robbert et al. revealed that PfpI exhibit both trypsin- and chymotrypsin-like specificities, whereas only several endopeptidase substrates (AAF-amc, LY-amc, and AFK-amc) were used to probe the cleavage preference. Considering that Hsp31, which shares a low sequence alignment but has similar catalytic triad with PH1704, exhibited aminopeptidase activity by preferring alanine and basic amino acids [5], [6], [7], [32], we speculated that PH1704 also hydrolyzes aminopeptidase substrates. Thus, distinctive amc-derived peptides were used to elucidate the substrate specificity of PH1704. Six substrates, including charged (L-R-amc and L-D-amc), neutral (L-A-amc and L-V-amc), bulky hydrophobic (L-F-amc), and polar (L-S-amc) types were selected for determining the specific activity. As shown in Fig. 4C, arginine, phenylalanine, alanine, valine, aspartate, and serine substrates are hydrolyzed with decreasing efficiency, in that order. The specific activity for hydrolyzing L-R-amc was 90 times to 300 times higher than those hydrolyzing other substrates. This finding suggests that PH1704 is an aminopeptidase with limited specificity, mainly cleaving after arginine and hardly cleaving other amino acids. Similar to Hsp31, PH1704 can function as an aminopeptidase [32]. However, the PH1704 also showed different substrate specificities and restrictive spectrum compared to Hsp31, which showed the highest activity with A-amc [32]. Considering that PfpI is an endopeptidase preferring basic and bulky hydrophobic amino acids in the P1 position (the group in the substrate binding to S1 pocket near the peptide bond of PH1704) and PH1704 can catalyze aminopeptidase substrates, we reassessed the function of PH1704 by determining both the aminopeptidase and endopeptidase activities. Thus, we selected four peptides (AF-amc, AAF-amc, AR-amc, and AAFR-amc) for further analysis according to the preference of PfpI. As an endopeptidase, the enzyme preferred the conversion of substrates with a positively charged (Arg) residue at the P1 site (Fig. 4C), which is a characteristic of cysteine protease [8], [9]. Compared with R-amc (100% activity), AAFR-amc was hydrolyzed with less efficiency (10%) (Fig. 4D). This observation strongly indicates that PH1704 exhibits primal aminopeptidase activity. We also tested the carboxypeptidase activity of PH1704 by using hippuryl-R as substrate and found no hydrolysis activity, which was consistent with the results of Hsp31. On the basis of the aforementioned results, we suggest that PH1704 be reannotated as an aminopeptidase with limited specificity and endopeptidase activity. The structural basis for its preference to the basic residues is presented in the subsequent section.

### SIFt pattern for predicting the binding mode of PH1704

**Figure 5:**
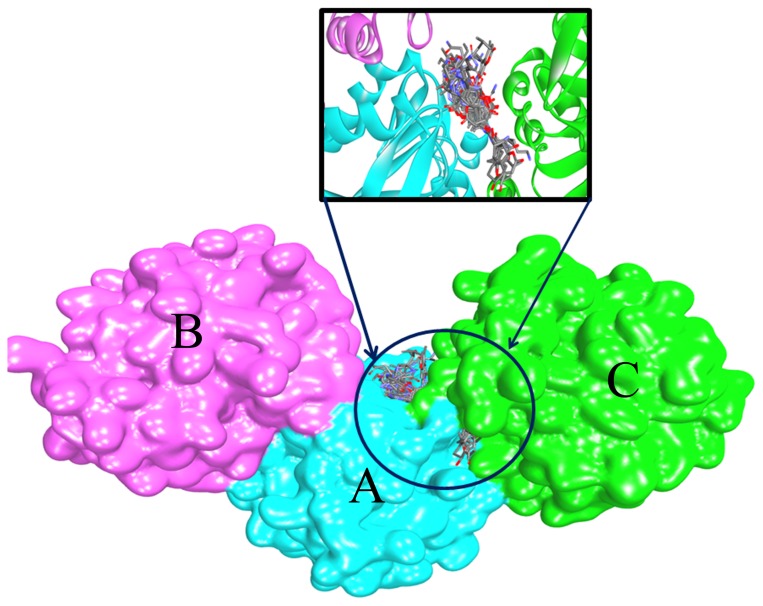
All 29 docked ligands located in AC contacts.

**Figure 6:**
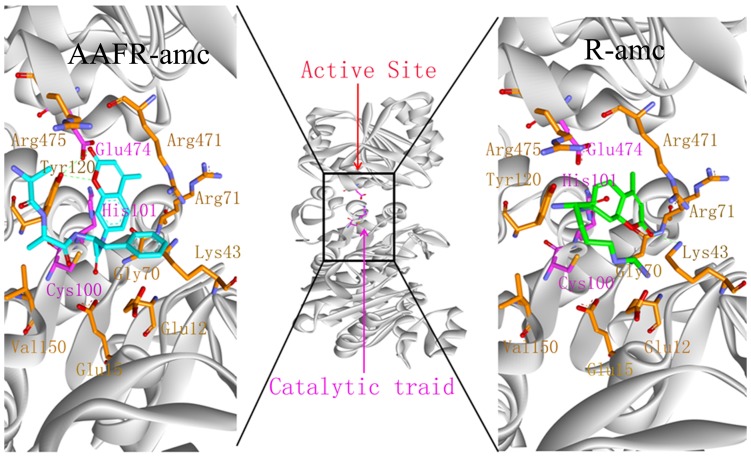
The active residues (Glu12, Glu15, Lys43, Gly70, Arg71, Cys100, His101, Tyr120, Val150, Arg471, Glu474, and Arg475) around the substrate, R-amc and AAFR-amc.

**Figure 7:**
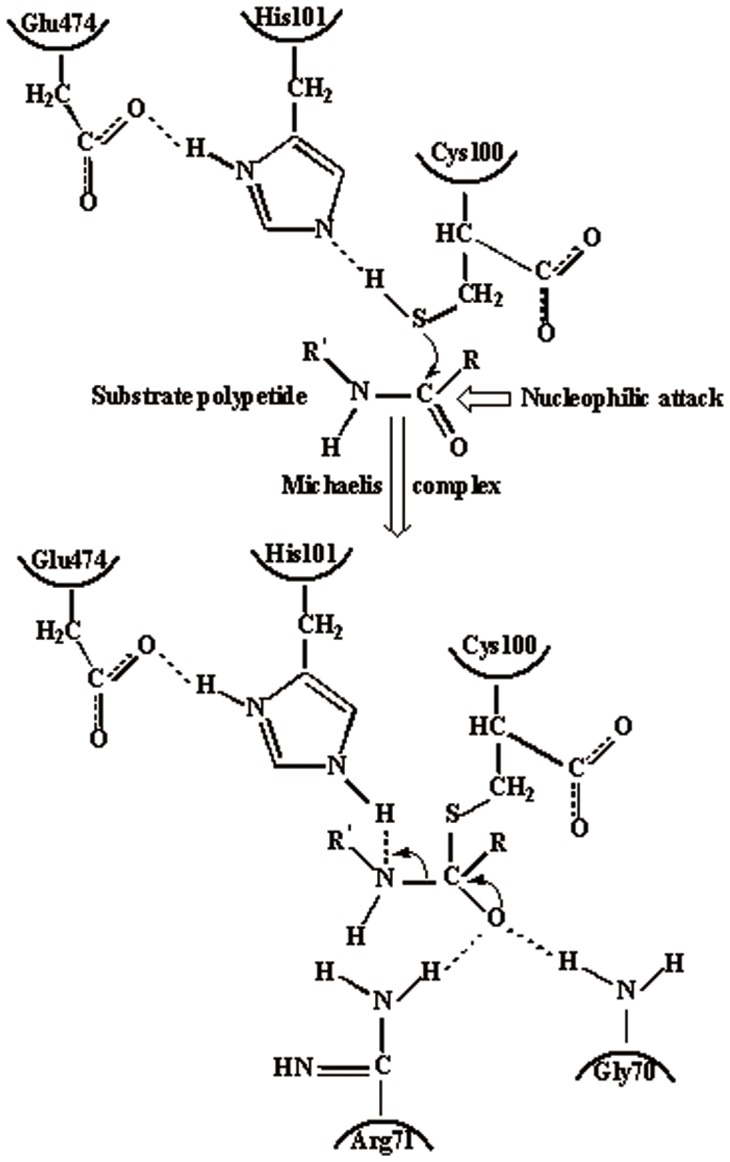
The mechanism for PH1704 is explained by the activated nucleophile (Cys100) attacking the hydroxyl carbon of the substrate to form a tetrahedral intermediate. His101 and Glu474 function as catalytic base.

Binding mode analysis for different molecules is used to determine the composition and the volume of the binding site. In a further study, 29 substrates were used to docking to PH1704 and PfpI with AutoDock 4.2. These substrates are as follows: succinyl-LLVY-amc, *t*-butyloxycarbonyl-LAR-amc, Ac-YVAD-amc, AAF-amc, ALK-amc, Ac-A-amc, L-A-amc, L-R-amc, L-D-amc, L-N-amc, L-G-amc, L-L-amc, L-K-amc, L-M-amc, L-F-amc, L-P-amc, L-T-amc, L-Y-amc, L-V-amc, L-S-amc, L-C-amc, L-H-amc, L-E-amc, L-I-amc, L-Q-amc, L-W-amc, AF-amc, AR-amc, and AAFR-amc. All ligands were docked in the AC contacts of two proteins (see from Fig. 5). All interpretations can be drawn based on Table 4. In summary, Glu12, Glu15, Lys43, Gly70, Arg71, Cys100, His101, Tyr120, Val150, Arg471, Glu474, and Arg475 (denoted to PH1704) are involved in substrate binding (Fig. 6). Meanwhile, Fig.6 showed the docked pose of L- R-amc and AAFR-amc, respectively. The 29 substrates were all in the AC contact and fit the active pocket well. Glu15, Gly70, and Cys100 are conservative in this DJ-1/ThiJ/PfpI superfamily (Fig. 1A), and the three residues may be identified in the evolutionary trace analysis of this superfamily. All ligands feature hydrophobic interactions with the sidechain of Lys43, His101, Tyr120, and Glu474 (any, sidechain, and hydrophobic). All ligands interact with the backbone and sidechain of Arg471. Some compounds (77%) also interact with the backbone of Arg71. All ligands feature aromatic and hydrophobic interactions with the Tyr120 side chain. Cys100, His101, and Glu474 function as a catalytic triad. From the sequence alignment, Gly70 is the oxygen hole that can back up the intermediates. In the docking study, 41% ligands forms a hydrogen bond between the hydroxyl oxygen group of substrates and the NH group of the Arg71 side chain (36% ligands form a hydrogen bond between the hydroxyl oxygen group of substrates and the NH group of the Arg471 sidechain). Arg71 also functions as an oxyion hole (Arg471 also acts as an oxyanion hole for the other catalytic triad (Cys500, His501, and Glu74). Thus, the mechanism for PH1704 is explained by the activated nucleophile (Cys100) attacking the hydroxyl carbon of the substrate to form a tetrahedral intermediate, which is stabilized by the NH group of Arg71 and Gly70 (Fig. 7). Glu12, Lys43, and Tyr120 interact with all compounds. And thence the three residues may be important in substrates binding. Our results are consistent with the experimental data [2]. The activity of E12T mutant is 3.8-fold higher than the wild type (WT) [2], [10]. The endopeptidase activity of the K43C mutant was 5.8-fold compared with the WT type [36]. In further studies, residue 120 was chosen for mutation.

**Table 4:** Averaged structural interaction fingerprints calculated over all successfully docked poses among two receptor conformations and 29 ligands presented for all identified interacting residues.

amino acid	any	backbone	side chain	polar	hydrophobic	H-bond acceptor	H-bond donor	aromatic	charged
E12	1	0	1	0	1	0	0	0	0
E15	0.3	0	0.32	0	0.32	0	0	0	0
K43	1	0.20	1	0	1	0	0	0	0
G70	1	1	0	0	1	0	0	0	0
R71	1	0.77	1	0.41	1	0.41	0	0	0
C100	0.9	0	0.96	0.14	1	0.14	0	0	0
H101	1	0	1	0.14	1	0.14	0	0	0
Y120	1	0	1	0	1	0	0	1	0
V150	0.4	0	0.41	0	0.41	0	0	0	0
R471	1	1	1	0.36	1	0.36	0	0	0
E474	1	0	1	0.16	1	0	0	0	0.16
R475	0.3	0.14	0.36	0	0.36	0	0	0	0

## Discussion

### Steady-state kinetics of Tyr120

PH1704 exhibits the hydrolytic activities of aminopeptidase and endopeptidase. The kinetic parameters *K*_cat_ and *K*_cat_/k_m_ with L-R-amc are 5.7- and 5-fold higher than that of AAFR-amc (Table 5), but *K*_m_ is similar for the two substrates. The small substrate (L-R-amc) can easily enter the substrate-binding pocket compared with the large substrate (AAFR-amc), thereby facilitating the enzyme nucleophilic attack. No marked difference was detected in the substrate affinity because the size of the substrate-binding cavity can accommodate AAFR-amc (Fig.2).

**Table 5:** Kinetic parameters for hydrolytic substrates of l-R-amc and L-AAFR-amc.

Enzyme	Aminopeptidase (R-AMC)		
	*k*_cat_ (min^−1^)	*K*_m_ (μM)	*k*_cat_/*K*_m_ (min^−1^μM ^−1^)
WT	0.646±0.05	12±0.65	0.052±0.008
Y120S	0.147±0.01	9.0±0.43	0.024±0.004
Y120W	1.2±0.07	20.6±0.77	0.066±0.005
Y120P	4.37±0.12	11.3±0.59	0.398±0.025
**Enzyme**	**Endopeptidase (AAFR-AMC)**		
	***k*_cat_ (min^−1^)**	***K*_m_ (μM)**	***k*_cat_/*K*_m_ (min^−1^μM ^−1^)**
WT	0.11±0.02	10±1.2	0.018±0.004
Y120S	0.084±0.01	6.5±0.4	0.017±0.003
Y120W	0.12±0.01	16.0±1.5	0.019±0.001
Y120P	1.13±0.07	5.4±0.3	0.21±0.03

Each experiment was repeated three times.

For the mutant Y120P with the aminopeptidase substrate L-R-amc,almost no change in the *K*_m_ of the WT and the Y120P mutant. The *K*_cat_ and K_cat_/*K*_m_ of the Y120P mutant were about 7 and 7.8 times higher than that of WT, respectively,indicating that the 120 site is involved in nucleophilic attack. The result showed an improvement in the catalytic efficiency of the Y120P mutant but no effect on substrate (AAFR-amc and L-R-amc) affinity in principle.

**Figure 8:**
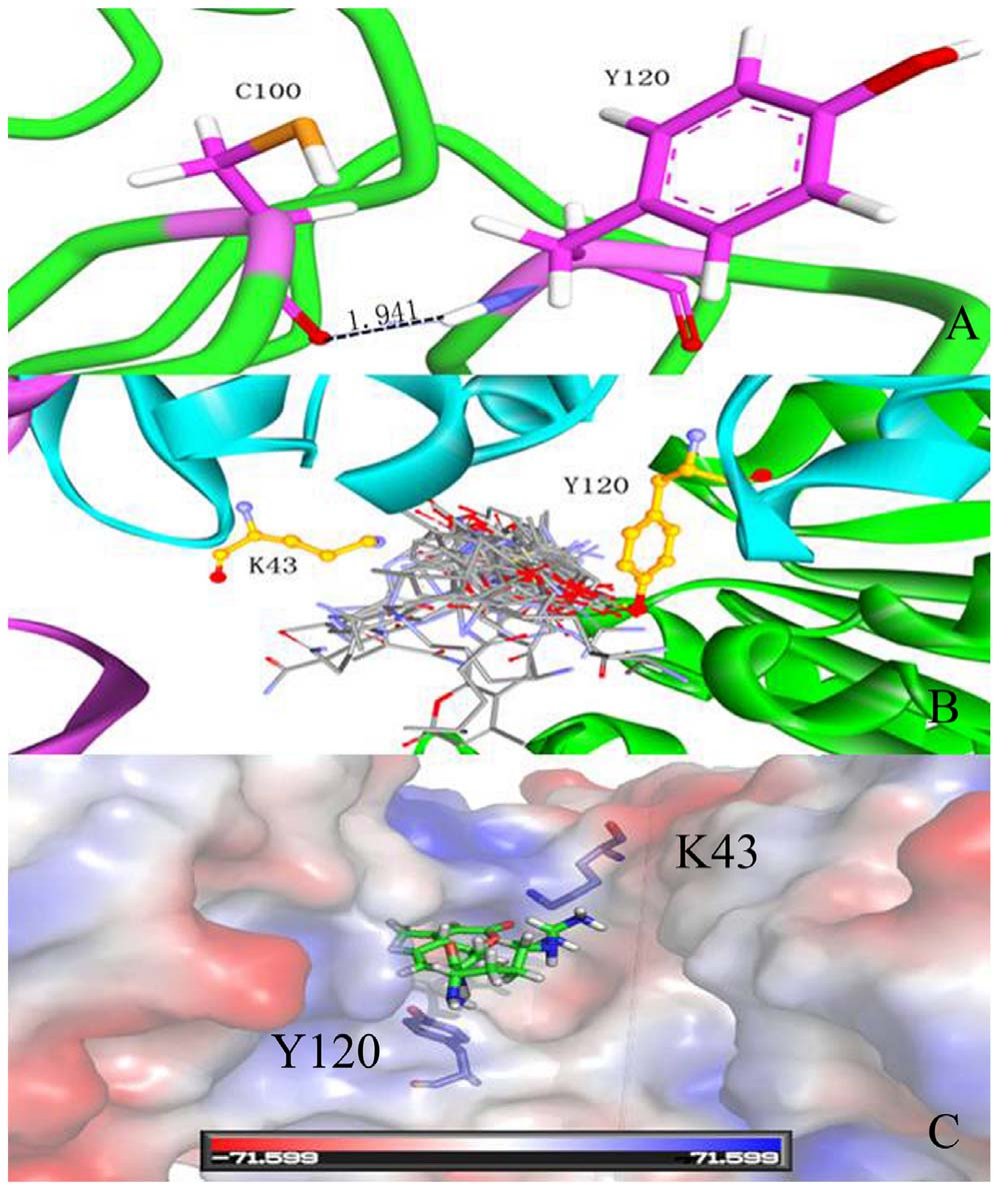
The dual-function of Tyr120: participation in enzyme nucleophilic catalytic through a hydrogen bond; acting as entrance gate with Lys43. A, hydrogen bond between Cys100 and Tyr120; B, 29 substrates docked in AC contacts; C, The entrance gate guide by Tyr120 and Lys43 (R-amc in the active sit).

The substrate R-AMC is small and can easily enter the substrate-binding pocket. Although the sidechain and the special hindrance decreased, no significant advantage was observed for the small molecular substrate, which did not affect the substrate binding of the WT and the Y120P mutant. As shown in Fig. 8A, a hydrogen bond is formed between the backchain of Cys100 and the NH group of the Tyr120 backchain (distance 2.0 Å). For the Y120P mutant, the hydrogen bond between Cys100 and Tyr120 is broken. The active pocket became flexible, which can facilitate the nucleophilic attack of group-SH. For the endopeptidase Y120P with AAFR-amc, the kinetic parameter *K*_m_ is 5.4 µM, which is half of that of the WT, whereas *K*_cat_ and *K*_cat_/k_m_ are 10- and 21-fold that of the WT. This result demonstrates a significant improvement in endopeptidase activity. As shown in Fig.8B, Tyr120 is located near the substrate-binding site. The substrate AAFR-amc is large, and the Arg sidechain does not fit well in the substrate-binding pocket. Thus, the nucleophilic attack of Cys100 was markedly influenced, and the catalytic efficiency decreased. However, for the Y120P mutant, the spacial steric hindrance of the sidechain of the benzene ring disappeared, which facilitated the entry of the large substrate to the catalytic center. The hydrogen bond between C100-Y120 disappeared, which benefited the catalytic attack of the active center Cys100. Thus, *K*_cat_ and *K*_cat_/*K*_m_ of the Y120P mutant improved significantly.

In summary, Tyr120 influenced enzyme catalysis, which regulated the catalytic conformation of Cys100 through a hydrogen bond, thereby affecting enzyme activity. No apparent change in enzyme activity was observed in Y120S and Y120W (Table 5) because the resistance of the hydrogen bond between Cys100 and the mutants remained. This result also indicates that Tyr120 participates in the nucleophilic catalysis. The aminopeptidase activity was higher than that of endopeptidase primarily because the aminopeptidase substrates are smaller. No change in enzyme activity was observed in the Y120W mutant, However, *K*_m_ was higer than that of the WT with both aminopeptidase and endopeptidase substrates. The sidechain group of Trp is larger than that of Tyr. Thus, the Y120W mutant prevents the substrate from entering pocket, resulting in lower substrate affinity.

### “Dual-function” of Tyr120

The “dual-function” of Tyr120 lies in its participation in enzyme nucleophilic catalytic through a hydrogen bond (Cys100-Y120). As shown in Fig. 1, only Hsp31 family class III has Tyr residue in position 120. Thus, Tyr120 is important in substrate binding in Hsp31 family class III. Tyr120 also functions as an entrance gate of the substrate with Lys43, as shown in Fig. 8B and 8C. Each substrate can enter the entrance gate to slide into the active site. Tyr120 and Lys43 act as the ceiling above the active site. When the ligand passed through this gate, the Tyr120 ring rotated nearly 360°and exhibited the π-π interaction with the indazole group of the substrates, which further hindered the unbinding process of substrates. The large group of Lys43 also blocked the substrates (the active pocket size of WT is 1622.1 Å^3^, calculated by CASTp server with 1.4 Å, whereas the active pocket size of the K43C mutant is 1670.5 Å^3^). Therefore, Tyr120 and Lys43 act as entrance gate regulators.

## Conclusion

The recombinant PH1704 protease from hyperthermophilic archaean *Pyrococcus horikoshii* OT3 is a member of the DJ-1/ThiJ/PfpI superfamily. Enzymatic properties showed that it was a thermophilic protease with optimal temperature 80°C and pH 8.0. The recombinant protein was a cysteine protease, existed in the form of dodecamer. It was identified as an aminopeptidase with limited substrate specificity that only prefers amc-linked substrate l-R-amc and an endopeptidase with lower activity. Structural analysis and experimental data showed that the residue Tyr120 participated in enzymatic catalysis, and acted as substrate entrance gate with Lys43. The results will be helpful for further modification of new enzyme.
